# Supplementary material for: Emerging Roles of Endocannabinoids as Key Lipid Mediators for a Successful Pregnancy
Source: Int J Mol Sci. 2023 Mar 9;24(6):5220. doi: 10.3390/ijms24065220 (PMC10048990; doi:10.3390/ijms24065220)
Supplement: Supplementary file 1 [file ijms-24-05220-s001.zip › ijms-2201277-supplementary.pdf]

Table S1. A summary of the main findings concerning the physiological role of the ECS in the early pregnancy processes (as discussed in paragraph 4).

Oviductal transport	***Species***	***Tissue, cells***	***Main findings***	***Ref.***
	Mus musculus (C57BL/6J)	oviduct, uterus, embryo	FAAH deficiency increases oviductal embryo retention by CB1-dependent signaling	[1]
	Mus musculus (C57BL/6J)	oviduct, uterus, embryo	CB1 deficiency and Meth-AEA increase oviductal embryo retention by CB1-dependent signaling	[2]
	Mus musculus (C57BL/6J)	oviduct, uterus, embryo	CB1/adrenergic signaling dysregulations increase oviductal embryo retention	[2]
	Mus musculus (C57BL/6J)	oviduct, uterus, embryo	Δ9-THC, but not CBD or CBN, increases oviductal embryo retention by CB1-dependent signaling	[1]
Blastocyst development	***Species***	***Tissue, cells***	***Main findings***	***Ref.***
	Mus musculus (C57BL/6J)	oviduct, uterus, embryo	FAAH deficiency impairs embryo development by CB1-dependent signaling	[1]
	Mus musculus (129/C57Bl6)	oviduct, uterus, embryo	CB1 and FAAH deficiency impairs embryo development	[3]
	Mus musculus (CD-1)	oviduct, uterus, embryo	CB1, CB2 and CB1/CB2 deficiency impairs embryo development	[4]
	Mus musculus (C57BL/6)	uterus, embryo	CB1/CB2 deficiency does not affect embryo development	[5]
	Mus Musculus (CD-1)	embryo culture	AEA and 2-AG inhibit the synchronous embryo development by CB1-dependent signaling.	[6]
	Mus musculus (C57BL/6J)	oviduct, uterus, embryo	Δ9-THC, but not CBD or CBN, impairs embryo transition to blastocyst by CB1-dependent signaling	[1]
	Mus Musculus (CD-1)	embryo culture	AEA, Δ9-THC, WIN55-212, CP 55,940, but not AA and CBD, impair embryo transition to blastocyst in a dose-dependent manner	[7]
Implantation	***Species***	***Tissue, cells***	***Main findings***	***Ref.***
	Mus musculus (CD-1)	uterus	AEA level is lower in receptive than non-receptive uteri	[4]
	Mus musculus (129/C57Bl6)	uterus	AEA and 2-AG levels are lower in IS than inter-IS	[8]
	Mus musculus (CD-1)	uterus	AEA level is lower in IS than inter-IS	[9]
	Mus musculus (CD-1)	uterus	NAPE-PLD activity is lower, while FAAH activity is higher, in IS than inter-IS	[10]
	Mus Musculus (CD-1)	uterus	NAPE-PLD activity and AEA levels are lower in IS than inter-IS and regulated by sex steroid hormones and blastocyst state.	[11]
	Mus musculus (CD-1)	embryo culture	AEA reduces blastocyst hatching by CB1-signaling	[9]
	Mus musculus (C57BL/6J)	uterus, embryo	FAAH deficiency impairs blastocyst implantation by CB1-dependent signaling	[1]
	Mus musculus (C57BL/6J)	uterus	CB1/CB2 deficiency impairs luminal epithelial organization and increases edema in the PDZ	[5]
	Mus musculus (CD-1)	embryo culture	CB1 is higher in dormant than activated blastocysts	[4]

	Mus musculus	embryo culture	CB1 is higher in dormant than activated blastocysts	[12]
	Mus musculus	embryo culture	AEA regulates blastocyst competency by differently modulating MAPK signaling and Ca^{2+} channel activity trough CB1-dependent mechanisms.	[12]
	Mus musculus (129/C57Bl6)	embryo, TSC culture	CB1 and FAAH deficiency affects cell migration-related genes in blastocysts and cell mobility in TSCs.	[3]
	Mus musculus (CD-1)	uterus, embryo	Δ9-THC impairs blastocyst implantation by CB1/CB2-dependent signaling	[4]
	Mus musculus (C57BL/6J)	uterus, embryo	Δ9-THC, but not CBD or CBN, impairs blastocyst implantation by CB1-dependent signaling	[1]
	Mus Musculus (CD-1)	uterus	Δ9-THC impairs blastocyst implantation by CB1-dependent signaling	[6]
	Mus musculus (CD-1)	uterus, embryo	CP 55,940 inhibits blastocyst implantation by CB1-dependent signaling	[9]
Decidualization	***Species***	***Tissue, cells***	***Main findings***	***Ref.***
	Homo sapiens	St-T1b cells and primary HdF cells	AEA impairs human ESC proliferation and differentiation, but not viability, by CB1-dependent signaling	[13]
	Homo sapiens	St-T1b cells and primary HdF cells	AEA impairs decidualization and reduces COX-2 level and PGE2 production by CB1-dependent signaling	[14]
	Homo sapiens	St-T1b cells	AEA induces apoptosis, ROS production, loss of ΔΨm, ER stress and reduces cell viability and proliferation partly by induction of COX-2 activity	[15]
	Homo sapiens	placental microsomes, St-T1b cells and primary HdF cells	AEA shows anti-aromatase activity and regulates aromatase expression and E2 secretion partly by CB1-dependent signaling	[16]
	Homo sapiens	ESC cells, BeWo cells, HTR8-SV/neo cells	Δ9-THC, CBD, CBN impair ESC decidualization, trophoblast-endometrium interaction and invasion	[17]
	Homo sapiens	placental microsomes, St-T1b cells and primary HdF cells	CBD, but not Δ9-THC, shows anti-aromatase activity and impairs decidualization in ESCs	[18]
	Homo sapiens	primary ESC and HdF cells	WIN55,212 inhibits decidual fibroblasts and ESC decidualization. WIN55,212 induces apoptosis and reduces intracellular cAMP levels	[19]
	Homo sapiens	St-T1b cells and primary HdF cells	WIN55,212, JWH-122 and UR-144 induce ROS/RNS production and ER stress. WIN55,212 reduces cell viability and proliferation, induces apoptosis and loss of ΔΨm partly by CB1-dependent signaling	[20]
	Homo sapiens, Mus musculus (C57BL/6)	uterus, primary mESCs, HdF	CB1/CB2 deficiency impairs decidualization, vascular remodeling and formation of avascular PDZ. CB1 deficiency impairs decidualization in mESCs and HdF.	[21]
	Rattus norvegicus (Wistar)	primary decidual cells	AEA induces apoptosis, loss of ΔΨm and ROS production by induction of COX-2 activity	[22]
	Rattus norvegicus (Wistar)	primary decidual cells	AEA reduces cell viability and induces apoptosis in decidual cells by CB1-dependent signaling	[23]
	Rattus norvegicus (Wistar)	primary decidual cells	AEA induces ROS production, loss of ΔΨm, and apoptosis in decidual cells by ceramide synthesis	[24]
	Rattus norvegicus (Wistar)	primary rat ESC; in vivo deciduoma model	AEA reduces cell viability and impairs differentiation of primary ESCs. AEA impairs decidualization and downregulates α2-MG, COX-2 and VEGF expression *in vivo*	[25]
	Rattus norvegicus	primary decidual cells	2-AG reduces cell viability and induces apoptosis in decidual cells by CB1-dependent signaling	[26]

	(Wistar)			
Placentation	***Species***	***Tissue, cells***	***Main findings***	***Ref.***
	Homo sapiens	BeWo cells	2-AG induces apoptosis, loss of ΔΨm, ROS/RNS production and reduces proliferation in BeWo cells by CB1 and/or CB2-dependent signaling.	[27]
	Homo sapiens	hCTs, BeWo cells	2-AG induces apoptosis, ROS/RNS production and ER stress partly by CB2-dependent signaling	[28]
	Homo sapiens	hCTs	2-AG impairs cAMP/PKA, p38 and ERK1/2 signaling, endocrine functions and placental protein expression, but not cell viability, in syncytiotrophoblasts by CB1 and/or CB2-dependent signaling	[29]
	Homo sapiens	hCTs	AEA induces apoptosis, loss of ΔΨm, ROS/RNS production, and intracellular Ca^{2+} level increase in hCTs by TRPV1-dependent mechanisms.	[30]
	Homo sapiens	BeWo cells	Meth-AEA impairs the syncytialization in Fsk-treated BeWo cells	[31]
	Homo sapiens	hCTs, BeWo cells	AEA reduces cell viability in hCTs and BeWo cells. AEA induces apoptosis, loss of ΔΨm and ROS/RNS production in BeWo cells by CB1 and/or CB2-dependent signaling	[32]
	Homo sapiens	HTR8-SV/neo cells	AEA and 2-AG modulate angiogenic factors partly via CB receptors. AEA and 2-AG promote HTR8-SV/neo tube formation, and 2-AG increase HTR8-SV/neo cells migration	[33]
	Homo sapiens	BeWo cells	JWH-018, JWH-122, UR-144 and Δ9-THC affect cell viability and cell proliferation. JWH-018, UR-144, JWH-122 induce ROS/RNS production; Δ9-THC, UR-144, JWH- 122 induce loss of ΔΨm. The effects on apoptosis are mediated by CB1 and/or CB2 dependent (for JWH-018, UR-144, Δ9-THC) or independent mechanisms (for JWH-122)	[34]
	Homo sapiens	hCTs, BeWo cells	WIN55,212 reduces cell viability in hCTs and cell viability and proliferation in BeWo cells. WIN55,212 reduces ΔΨm and increases apoptosis by CB1-dependent mechanisms in BeWo cells.	[35]
	Homo sapiens	BeWo cells	Δ9-THC impairs syncytialization and biochemical differentiation, endocrine secretion, mitochondrial dynamics partly by CB1 and CB2-dependent signaling. Δ9-THC increases stress responses, intracellular defenses, ROS production and reduces ΔΨm and oxygen consumption	[36]
	Homo sapiens	HTR8-SV/neo cells	Δ9-THC impairs trophoblast invasive capacity and mitochondrial dynamics through CB1 and/or CB2 receptors. Δ9-THC increases ROS production and stress responses, and decreases mitochondrial respiration and ΔΨm.	[37]
	Homo sapiens	BeWo cells	Δ9-THC induces ER stress through CB1 and CB2 receptors. Δ9-THC reduces mitochondrial respiration and ATP-coupling efficiency.	[38]
	Homo sapiens	chorionic villous explants cultures, BeWo cells	Δ9-THC upregulates aromatase and ER*α* expression by CB1 and CB2-dependent signaling	[39]
	Homo sapiens	BeWo cells	Δ9-THC reduces GLU1 and GR expression in BeWo cells.	[40]
	Homo sapiens	placenta, HUVECs	*Cannabis* exposure leads to placenta vascular defects. Δ9-THC reduces HUVEC proliferation, migration, tube formation capacity. Δ9-THC inhibits RhoA/MLC signaling pathway affecting cell migration and tube formation.	[41]
	Homo sapiens	BeWo cells, HTR8-SV/neo cells	CBD reduces cell viability, proliferation, ΔΨm, and increases apoptosis in BeWo cells and HTR8-SV/neo cells. No effects on ROS/RNS production. CBD increases autophagy by HIF1*α* and impairs migration in HTR8-SV/neo cells.	[42]
	Homo sapiens	placenta, hCTs	hCTs express CNR1 and show higher levels of 2-AG than AEA. CB1 is involved in hCT invasion	[43]

Homo sapiens	BeWo cells	AEA and Δ9-THC modulate FA uptake in time, dose and pH-dependent manner. The acute modulatory effects of AEA and Δ9-THC are not mediated by CB1 or CB2-dependent signaling	[44]
Homo sapiens	placenta, BeWo cells	AEA downregulates syncytialization-related genes and BCPR transporter expression by CB2/cAMP signaling. AEA is not a substrate or inhibitor of BCRP in BeWo cells.	[45]
Homo sapiens	placenta, BeWo cells	CBD inhibits the BCRP efflux activity both in BeWo cells and perfused placentas as *ex vivo* models. CBD does not affect placental tissues viability.	[46]
Macaca mulatta (rhesus macaque)	placenta	Δ9-THC reduces the amniotic fluid volume, maternal perfusion of the IVS and fetal oxygen availability. Δ9-THC alters histology and gene expression profile in placenta, with DEGs enrichment in cytokine binding, regulation of cell migration, cell-substrate adhesion, angiogenesis, and vascular development	[47]
Rattus norvegicus (Wistar)	placenta	Δ9-THC exposure results in symmetrical IUGR, increased placental weights, and reduced fetal:placental weight ratio. Δ9-THC exposure results in an increased labyrinth layer areas, maternal/fetal blood space ratio and number of pericytes. Δ9-THC reduces GLU1 and GR expression.	[40]
Mus musculus (C57BL/6)	placenta	Δ9-THC attenuates placental angiogenesis by inhibiting RhoA/ MLC signaling pathway	[41]
Mus musculus (C57BL/6)	uterus, TS cells	PCP signaling in cooperation with CB1 receptors regulates trophoblast migration in placentation	[48]
Mus musculus (C57BL/6J)	oviduct, uterus, placenta	CB1 deficient mice show compromised placentation, reduced fetal trophoblast proliferation and invasiveness	[49]

Abbreviations: 2-AG, 2-arachidonoylglycerol; AEA, anandamide; BCRP, Breast cancer resistance protein; cAMP/PKA, cyclic Adenosine monophosphate/protein kinase A; CB, G protein-coupled cannabinoid receptors; CBD, cannabidiol; CBN, Cannabinol; COX-2, Cyclooxygenase-2; DEG, differentially expressed genes; E2, estradiol; ECS, endocannabinoid system; ER, endoplasmic reticulum; ERα, estrogen receptor α; ESC, endometrial stromal cells; FA, acid folic; FAAH, fatty acid amide hydrolase; Fsk, forskolin; GLU1, glucose transporter 1; GR, glucocorticoid receptors; hCTs, human cytotrophoblast cells; HdFs, human decidual fibroblasts; HIF1α, hypoxia-inducible factor; hTS, human trophoblast cells; HUVECs, human vein endothelial cells; IS, implantation sites; IUGR, intrauterine growth restriction; IVS, intervillous space; MAPK, mitogen-activated protein kinase; Meth-AEA, methanandamide; NAPE-PLD; N-acylphosphatidylethanolamine specific phospholipase d-like; PCP, planar cell polarity; PDZ, primary decidua zone; PGE, prostaglandin E; RhoA/MLC, Ras homolog family member A/myosin light chain; ROS/RNS, reactive oxygen species/reactive nitrogen species; St-T1b, human immortalized endometrial stromal cell line; TRPV1, transient receptor potential vanilloid 1; TSC, trophoblast stem cells; VEGF, vascular endothelial growth factor; α2-M, α2-macroglobulin; Δ9-THC, Δ9-tetrahydrocannabinol; ΔΨm, mitochondrial membrane potential.

References

1. Wang, H.; Xie, H.; Guo, Y.; Zhang, H.; Takahashi, T.; Kingsley, P. J.; Marnett, L. J.; Das, S. K.; Cravatt, B. F.; Dey, S. K., Fatty acid amide hydrolase deficiency limits early pregnancy events. *J Clin Invest* **2006,** 116, (8), 2122-31.
2. Wang, H.; Guo, Y.; Wang, D.; Kingsley, P. J.; Marnett, L. J.; Das, S. K.; DuBois, R. N.; Dey, S. K., Aberrant cannabinoid signaling impairs oviductal transport of embryos. *Nat Med* **2004,** 10, (10), 1074-80.
3. Xie, H.; Sun, X.; Piao, Y.; Jegga, A. G.; Handwerger, S.; Ko, M. S.; Dey, S. K., Silencing or amplification of endocannabinoid signaling in blastocysts via CB1 compromises trophoblast cell migration. *The Journal of biological chemistry* **2012,** 287, (38), 32288-97.
4. Paria, B. C.; Song, H.; Wang, X.; Schmid, P. C.; Krebsbach, R. J.; Schmid, H. H.; Bonner, T. I.; Zimmer, A.; Dey, S. K., Dysregulated cannabinoid signaling disrupts uterine receptivity for embryo implantation. *J Biol Chem* **2001,** 276, (23), 20523-8.
5. Li, Y.; Bian, F.; Sun, X.; Dey, S. K., Mice Missing Cnr1 and Cnr2 Show Implantation Defects. *Endocrinology* **2019,** 160, (4), 938-946.
6. Paria, B. C.; Ma, W.; Andrenyak, D. M.; Schmid, P. C.; Schmid, H. H.; Moody, D. E.; Deng, H.; Makriyannis, A.; Dey, S. K., Effects of cannabinoids on preimplantation mouse embryo development and implantation are mediated by brain-type cannabinoid receptors. *Biology of reproduction* **1998,** 58, (6), 1490-5.
7. Paria, B. C.; Das, S. K.; Dey, S. K., The preimplantation mouse embryo is a target for cannabinoid ligand-receptor signaling. *Proceedings of the National Academy of Sciences of the United States of America* **1995,** 92, (21), 9460-4.
8. Wang, H.; Xie, H.; Sun, X.; Kingsley, P. J.; Marnett, L. J.; Cravatt, B. F.; Dey, S. K., Differential regulation of endocannabinoid synthesis and degradation in the uterus during embryo implantation. *Prostaglandins Other Lipid Mediat* **2007,** 83, (1-2), 62-74.
9. Schmid, P. C.; Paria, B. C.; Krebsbach, R. J.; Schmid, H. H.; Dey, S. K., Changes in anandamide levels in mouse uterus are associated with uterine receptivity for embryo implantation. *Proceedings of the National Academy of Sciences of the United States of America* **1997,** 94, (8), 4188-92.
10. Paria, B. C.; Deutsch, D. D.; Dey, S. K., The uterus is a potential site for anandamide synthesis and hydrolysis: differential profiles of anandamide synthase and hydrolase activities in the mouse uterus during the periimplantation period. *Mol Reprod Dev* **1996,** 45, (2), 183-92.

11. Guo, Y.; Wang, H.; Okamoto, Y.; Ueda, N.; Kingsley, P. J.; Marnett, L. J.; Schmid, H. H.; Das, S. K.; Dey, S. K., N-acylphosphatidylethanolamine-hydrolyzing phospholipase D is an important determinant of uterine anandamide levels during implantation. *J Biol Chem* **2005,** 280, (25), 23429-32.
12. Wang, H.; Matsumoto, H.; Guo, Y.; Paria, B. C.; Roberts, R. L.; Dey, S. K., Differential G protein-coupled cannabinoid receptor signaling by anandamide directs blastocyst activation for implantation. *Proceedings of the National Academy of Sciences of the United States of America* **2003,** 100, (25), 14914-9.
13. Almada, M.; Amaral, C.; Diniz-da-Costa, M.; Correia-da-Silva, G.; Teixeira, N. A.; Fonseca, B. M., The endocannabinoid anandamide impairs in vitro decidualization of human cells. *Reproduction* **2016,** 152, (4), 351-61.
14. Almada, M.; Cunha, S.; Fonseca, B. M.; Amaral, C.; Piscitelli, F.; Di Marzo, V.; Correia-da-Silva, G.; Teixeira, N., Anandamide interferes with human endometrial stromal-derived cell differentiation: An effect dependent on inhibition of cyclooxygenase-2 expression and prostaglandin E2 release. *Biofactors* **2016,** 42, (3), 277-86.
15. Almada, M.; Fonseca, B. M.; Amaral, C.; Diniz-da-Costa, M.; Correia-da-Silva, G.; Teixeira, N., Anandamide oxidative metabolism-induced endoplasmic reticulum stress and apoptosis. *Apoptosis : an international journal on programmed cell death* **2017,** 22, (6), 816-826.
16. Almada, M.; Oliveira, A.; Amaral, C.; Fernandes, P. A.; Ramos, M. J.; Fonseca, B.; Correia-da-Silva, G.; Teixeira, N., Anandamide targets aromatase: A breakthrough on human decidualization. *Biochim Biophys Acta Mol Cell Biol Lipids* **2019,** 1864, (12), 158512.
17. Neradugomma, N. K.; Drafton, K.; Mor, G. G.; Mao, Q., Marijuana-derived cannabinoids inhibit uterine endometrial stromal cell decidualization and compromise trophoblast-endometrium cross-talk. *Reprod Toxicol* **2019,** 87, 100-107.
18. Almada, M.; Amaral, C.; Oliveira, A.; Fernandes, P. A.; Ramos, M. J.; Fonseca, B. M.; Correia-da-Silva, G.; Teixeira, N., Cannabidiol (CBD) but not tetrahydrocannabinol (THC) dysregulate in vitro decidualization of human endometrial stromal cells by disruption of estrogen signaling. *Reprod Toxicol* **2020,** 93, 75-82.
19. Moghadam, K. K.; Kessler, C. A.; Schroeder, J. K.; Buckley, A. R.; Brar, A. K.; Handwerger, S., Cannabinoid receptor I activation markedly inhibits human decidualization. *Mol Cell Endocrinol* **2005,** 229, (1-2), 65-74.
20. Fonseca, B. M.; Fernandes, R.; Almada, M.; Santos, M.; Carvalho, F.; Teixeira, N. A.; Correia-da-Silva, G., Synthetic cannabinoids and endometrial stromal cell fate: Dissimilar effects of JWH-122, UR-144 and WIN55,212-2. *Toxicology* **2019,** 413, 40-47.
21. Li, Y.; Dewar, A.; Kim, Y. S.; Dey, S. K.; Sun, X., Pregnancy success in mice requires appropriate cannabinoid receptor signaling for primary decidua formation. *Elife* **2020,** 9.
22. Almada, M.; Piscitelli, F.; Fonseca, B. M.; Di Marzo, V.; Correia-da-Silva, G.; Teixeira, N., Anandamide and decidual remodelling: COX-2 oxidative metabolism as a key regulator. *Biochim Biophys Acta* **2015,** 1851, (11), 1473-81.
23. Fonseca, B. M.; Correia-da-Silva, G.; Teixeira, N. A., Anandamide-induced cell death: dual effects in primary rat decidual cell cultures. *Placenta* **2009,** 30, (8), 686-92.
24. Fonseca, B. M.; Correia-da-Silva, G.; Teixeira, N. A., The endocannabinoid anandamide induces apoptosis of rat decidual cells through a mechanism involving ceramide synthesis and p38 MAPK activation. *Apoptosis* **2013,** 18, (12), 1526-35.
25. Fonseca, B. M.; Correia-da-Silva, G.; Teixeira, N. A., Anandamide restricts uterine stromal differentiation and is critical for complete decidualization. *Mol Cell Endocrinol* **2015,** 411, 167-76.
26. Fonseca, B. M.; Correia-da-Silva, G.; Taylor, A. H.; Lam, P. M.; Marczylo, T. H.; Bell, S. C.; Konje, J. C.; Teixeira, N. A., The endocannabinoid 2-arachidonoylglycerol (2-AG) and metabolizing enzymes during rat fetoplacental development: a role in uterine remodelling. *The international journal of biochemistry & cell biology* **2010,** 42, (11), 1884-92.
27. Costa, M. A.; Fonseca, B. M.; Keating, E.; Teixeira, N. A.; Correia-da-Silva, G., 2-arachidonoylglycerol effects in cytotrophoblasts: metabolic enzymes expression and apoptosis in BeWo cells. *Reproduction* **2014,** 147, (3), 301-11.
28. Almada, M.; Costa, L.; Fonseca, B.; Alves, P.; Braga, J.; Goncalves, D.; Teixeira, N.; Correia-da-Silva, G., The endocannabinoid 2-arachidonoylglycerol promotes endoplasmic reticulum stress in placental cells. *Reproduction* **2020,** 160, (2), 171-180.
29. Costa, M. A.; Fonseca, B. M.; Mendes, A.; Braga, J.; Teixeira, N. A.; Correia-da-Silva, G., The endocannabinoid 2-arachidonoylglycerol dysregulates the synthesis of proteins by the human syncytiotrophoblast. *Biochim Biophys Acta* **2016,** 1861, (3), 205-12.
30. Costa, M. A.; Fonseca, B. M.; Keating, E.; Teixeira, N. A.; Correia-da-Silva, G., Transient receptor potential vanilloid 1 is expressed in human cytotrophoblasts: induction of cell apoptosis and impairment of syncytialization. *The international journal of biochemistry & cell biology* **2014,** 57, 177-85.
31. Etcheverry, T.; Accialini, P.; Palligas, M.; Loureiro, F.; Saraco, N.; Martinez, N.; Farina, M., Endocannabinoid signaling impairs syncytialization: Using flow cytometry to evaluate forskolin-induced cell fusion. *Placenta* **2021,** 103, 152-155.
32. Costa, M. A.; Fonseca, B. M.; Teixeira, N. A.; Correia-da-Silva, G., The endocannabinoid anandamide induces apoptosis in cytotrophoblast cells: involvement of both mitochondrial and death receptor pathways. *Placenta* **2015,** 36, (1), 69-76.
33. Maia, J.; Fonseca, B. M.; Teixeira, N.; Correia-da-Silva, G., The endocannabinoids anandamide and 2-arachidonoylglycerol modulate the expression of angiogenic factors on HTR8/SVneo placental cells. *Prostaglandins Leukot Essent Fatty Acids* **2022,** 180, 102440.
34. Almada, M.; Alves, P.; Fonseca, B. M.; Carvalho, F.; Queiros, C. R.; Gaspar, H.; Amaral, C.; Teixeira, N. A.; Correia-da-Silva, G., Synthetic cannabinoids JWH-018, JWH-122, UR-144 and the phytocannabinoid THC activate apoptosis in placental cells. *Toxicol Lett* **2020,** 319, 129-137.
35. Almada, M.; Costa, L.; Fonseca, B. M.; Amaral, C.; Teixeira, N.; Correia-da-Silva, G., The synthetic cannabinoid WIN-55,212 induced-apoptosis in cytotrophoblasts cells by a mechanism dependent on CB1 receptor. *Toxicology* **2017,** 385, 67-73.
36. Walker, O. S.; Ragos, R.; Gurm, H.; Lapierre, M.; May, L. L.; Raha, S., Delta-9-tetrahydrocannabinol disrupts mitochondrial function and attenuates syncytialization in human placental BeWo cells. *Physiol Rep* **2020,** 8, (13), e14476.
37. Walker, O. S.; Gurm, H.; Sharma, R.; Verma, N.; May, L. L.; Raha, S., Delta-9-tetrahydrocannabinol inhibits invasion of HTR8/SVneo human extravillous trophoblast cells and negatively impacts mitochondrial function. *Sci Rep* **2021,** 11, (1), 4029.

38. Lojpur, T.; Easton, Z.; Raez-Villanueva, S.; Laviolette, S.; Holloway, A. C.; Hardy, D. B., Delta9-Tetrahydrocannabinol leads to endoplasmic reticulum stress and mitochondrial dysfunction in human BeWo trophoblasts. *Reprod Toxicol* **2019,** 87, 21-31.
39. Maia, J.; Almada, M.; Midao, L.; Fonseca, B. M.; Braga, J.; Goncalves, D.; Teixeira, N.; Correia-da-Silva, G., The Cannabinoid Delta-9-tetrahydrocannabinol Disrupts Estrogen Signaling in Human Placenta. *Toxicol Sci* **2020,** 177, (2), 420-430.
40. Natale, B. V.; Gustin, K. N.; Lee, K.; Holloway, A. C.; Laviolette, S. R.; Natale, D. R. C.; Hardy, D. B., Delta9-tetrahydrocannabinol exposure during rat pregnancy leads to symmetrical fetal growth restriction and labyrinth-specific vascular defects in the placenta. *Sci Rep* **2020,** 10, (1), 544.
41. Chang, X.; Li, H.; Li, Y.; He, Q.; Yao, J.; Duan, T.; Wang, K., RhoA/MLC signaling pathway is involved in Delta(9)-tetrahydrocannabinol-impaired placental angiogenesis. *Toxicol Lett* **2018,** 285, 148-155.
42. Alves, P.; Amaral, C.; Teixeira, N.; Correia-da-Silva, G., Cannabidiol disrupts apoptosis, autophagy and invasion processes of placental trophoblasts. *Arch Toxicol* **2021,** 95, (10), 3393-3406.
43. Gormley, M.; Oliverio, O.; Kapidzic, M.; Ona, K.; Hall, S.; Fisher, S. J., RNA profiling of laser microdissected human trophoblast subtypes at mid-gestation reveals a role for cannabinoid signaling in invasion. *Development* **2021,** 148, (20).
44. Araujo, J. R.; Goncalves, P.; Martel, F., Effect of cannabinoids upon the uptake of folic acid by BeWo cells. *Pharmacology* **2009,** 83, (3), 170-6.
45. Szilagyi, J. T.; Composto-Wahler, G. M.; Joseph, L. B.; Wang, B.; Rosen, T.; Laskin, J. D.; Aleksunes, L. M., Anandamide down-regulates placental transporter expression through CB2 receptor-mediated inhibition of cAMP synthesis. *Pharmacol Res* **2019,** 141, 331-342.
46. Feinshtein, V.; Erez, O.; Ben-Zvi, Z.; Eshkoli, T.; Sheizaf, B.; Sheiner, E.; Holcberg, G., Cannabidiol enhances xenobiotic permeability through the human placental barrier by direct inhibition of breast cancer resistance protein: an ex vivo study. *Am J Obstet Gynecol* **2013,** 209, (6), 573 e1-573 e15.
47. Roberts, V. H. J.; Schabel, M. C.; Boniface, E. R.; D'Mello, R. J.; Morgan, T. K.; Terrobias, J. J. D.; Graham, J. A.; Borgelt, L. M.; Grant, K. A.; Sullivan, E. L.; Lo, J. O., Chronic prenatal delta-9-tetrahydrocannabinol exposure adversely impacts placental function and development in a rhesus macaque model. *Sci Rep* **2022,** 12, (1), 20260.
48. Kim, Y. S.; Li, Y.; Yuan, J.; Borg, J. P.; Sun, X.; Dey, S. K., Cannabinoid and planar cell polarity signaling converges to direct placentation. *Proceedings of the National Academy of Sciences of the United States of America* **2021,** 118, (38).
49. Sun, X.; Xie, H.; Yang, J.; Wang, H.; Bradshaw, H. B.; Dey, S. K., Endocannabinoid signaling directs differentiation of trophoblast cell lineages and placentation. *Proceedings of the National Academy of Sciences of the United States of America* **2010,** 107, (39), 16887-92.
